# Persistent cellular immunity to SARS-CoV-2 infection

**DOI:** 10.1084/jem.20202515

**Published:** 2021-01-27

**Authors:** Gaëlle Breton, Pilar Mendoza, Thomas Hägglöf, Thiago Y. Oliveira, Dennis Schaefer-Babajew, Christian Gaebler, Martina Turroja, Arlene Hurley, Marina Caskey, Michel C. Nussenzweig

**Affiliations:** 1 Laboratory of Molecular Immunology, The Rockefeller University, New York, NY; 2 Hospital Program Direction, The Rockefeller University, New York, NY; 3 Howard Hughes Medical Institute, Baltimore, MD

## Abstract

SARS-CoV-2 is responsible for an ongoing pandemic that has affected millions of individuals around the globe. To gain further understanding of the immune response in recovered individuals, we measured T cell responses in paired samples obtained an average of 1.3 and 6.1 mo after infection from 41 individuals. The data indicate that recovered individuals show persistent polyfunctional SARS-CoV-2 antigen–specific memory that could contribute to rapid recall responses. Recovered individuals also show enduring alterations in relative overall numbers of CD4^+^ and CD8^+^ memory T cells, including expression of activation/exhaustion markers, and cell division.

## Teaser

We show that SARS-CoV-2 infection elicits broadly reactive and highly functional T cell responses. In addition, recovered individuals show persistent alterations in CD4^+^ and CD8^+^ T cell memory compartments 6 months after infection.

## Introduction

Individuals who are infected with SARS-CoV-2 develop both cellular and humoral immune responses to the virus (Robbiani et al., 2020; Gaebler et al., 2020* Preprint*; Suthar et al., 2020; Ni et al., 2020; Grifoni et al., 2020; Peng et al., 2020; Sekine et al., 2020; Rydyznski Moderbacher et al., 2020; Braun et al., 2020; Zhou et al., 2020). Antibody responses develop to most of the structural proteins expressed by the virus, and nearly all individuals develop neutralizing antibodies directed to the receptor binding domain of the Spike trimer (S; Robbiani et al., 2020; Gaebler et al., 2020* Preprint*; Suthar et al., 2020; Ni et al., 2020; Lumley et al., 2020 *Preprint*). Cellular immune responses can be broad but vary widely (Grifoni et al., 2020; Peng et al., 2020; Sekine et al., 2020; Rydyznski Moderbacher et al., 2020; Braun et al., 2020; Zhou et al., 2020), and lymphopenia is a prominent feature of more severe infection, affecting CD4^+^ and CD8^+^ T cells, as well as B cells (Tavakolpour et al., 2020; Chen and John Wherry, 2020; Huang et al., 2020; Giamarellos-Bourboulis et al., 2020; Tan et al., 2020; Kuri-Cervantes et al., 2020; Mathew et al., 2020).

Careful studies of immune phenotypes in moderate, severe, and recovered individuals revealed T cell responses ranging from undetectable to robust CD8^+^ T cell and/or CD4^+^ T cell activation and proliferation (Grifoni et al., 2020; Peng et al., 2020; Braun et al., 2020; Neidleman et al., 2020 *Preprint*). During acute infection, T cells displayed a highly activated cytotoxic phenotype, whereas convalescent patients harbored polyfunctional SARS-CoV-2–specific T cells that display a stem-like memory phenotype (Sekine et al., 2020; Neidleman et al., 2020 *Preprint*; Weiskopf et al., 2020). In addition, cross-reactivity with seasonal/common cold coronaviruses has been reported, suggesting that these responses may be associated with a milder clinical course (Sekine et al., 2020; Le Bert et al., 2020; Mateus et al., 2020).

Coronaviruses elicit variable levels of persistent immunity. For example, individuals infected with Middle East respiratory syndrome remain immune for 1–3 yr, while protection from seasonal coronaviruses is short-lived (Wu et al., 2007; Edridge et al., 2020; Tang et al., 2011). Although there is increasing evidence that cellular immunity plays a major role in resolution of COVID-19, little is known about the persistence of cellular immunity to SARS-CoV-2 (Rodda et al., 2021; Rydyznski Moderbacher et al., 2020). This is a particularly important issue when considering an individual’s ability to resist a second exposure to the virus.

To determine whether cellular immunity to SARS-CoV-2 persists half a year after infection, we examined paired samples obtained an average of 1.3 and 6.1 mo after infection from a cohort of 41 COVID-19–convalescent volunteers (Robbiani et al., 2020; Gaebler et al., 2020* Preprint*). All of the individuals tested had RT-PCR–confirmed SARS-CoV-2 infection or were close contacts who had seroconverted and were RT-PCR negative at the second time point (Gaebler et al., 2020* Preprint*). The cohort was 63.4% male and 36.6% female, 24–73 yr old, and skewed to Caucasians with mild forms of the disease, with only 8 out of the 41 requiring hospitalization (Table S1).

## Results

### Changes in circulating T cells after COVID-19

**Figure 1.**
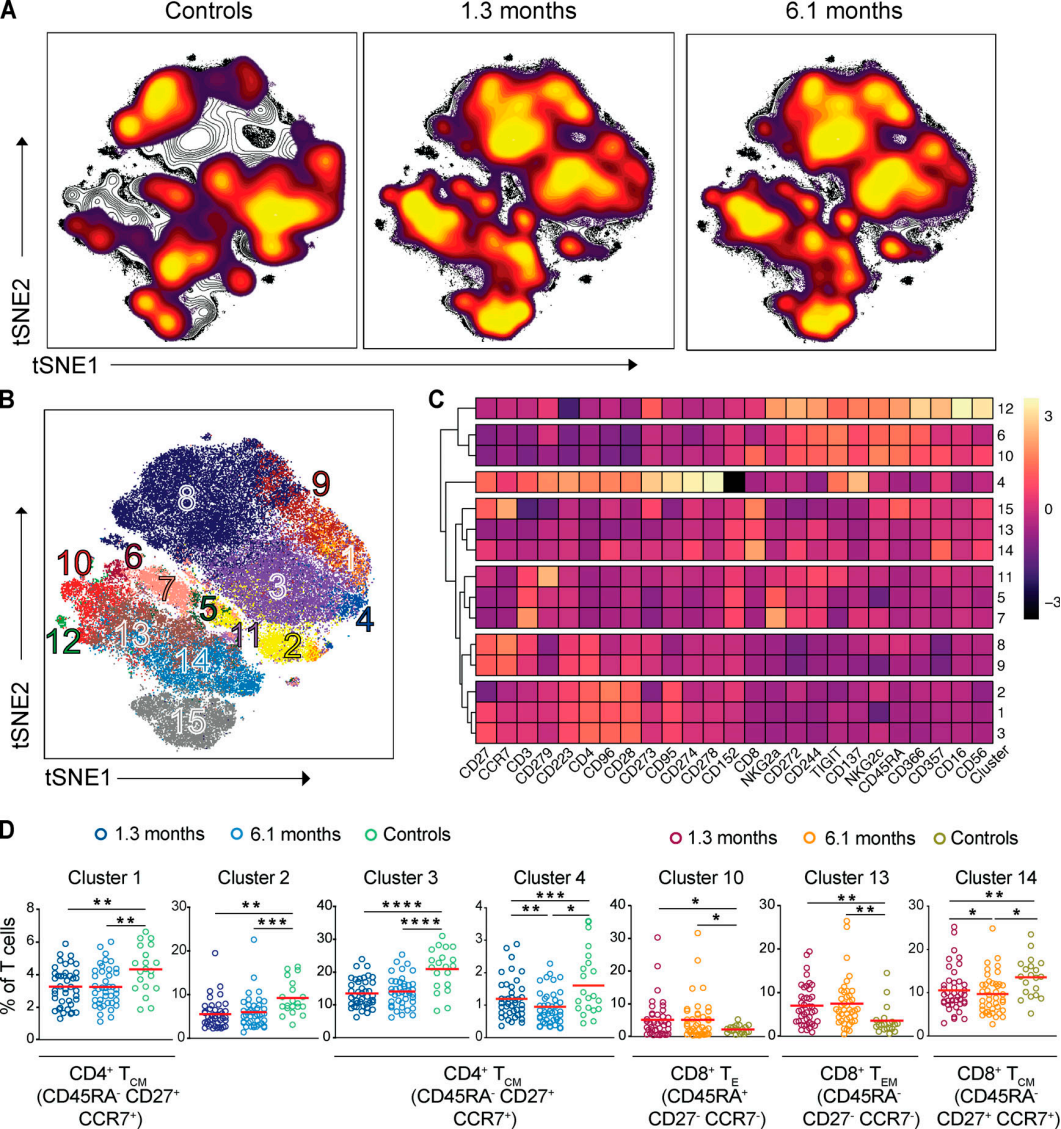
**Persistent longitudinal changes in the phenotypic landscape of T cells in individuals recovered from COVID-19.** **(A)** Global viSNE projection of pooled T cells for all participants pooled (controls, *n* = 20; COVID-19–convalescent individuals, *n* = 41) shown in background contour plots, with overlaid projections of concatenated controls, convalescent patients at 1.3 mo, and convalescent patients at 6.1 mo, respectively. **(B)** viSNE projection of pooled T cells for all participants of T cell clusters identified by FlowSOM clustering. **(C)** Column-scaled *z*-scores of MFI as indicated by cluster and marker. **(D)** Frequency of T cells from each group in FlowSOM clusters indicated. Each dot represents an individual with COVID-19 at 1.3 mo (dark blue for CD4^+^ T cells and dark red for CD8^+^ T cells) or 6.1 mo (light blue for CD4^+^ T cells and orange for CD8^+^ T cells) as well as control individuals (green). *, P < 0.05; **, P < 0.01; ***, P < 0.001; ****, P < 0.0001.

**Figure S1.**
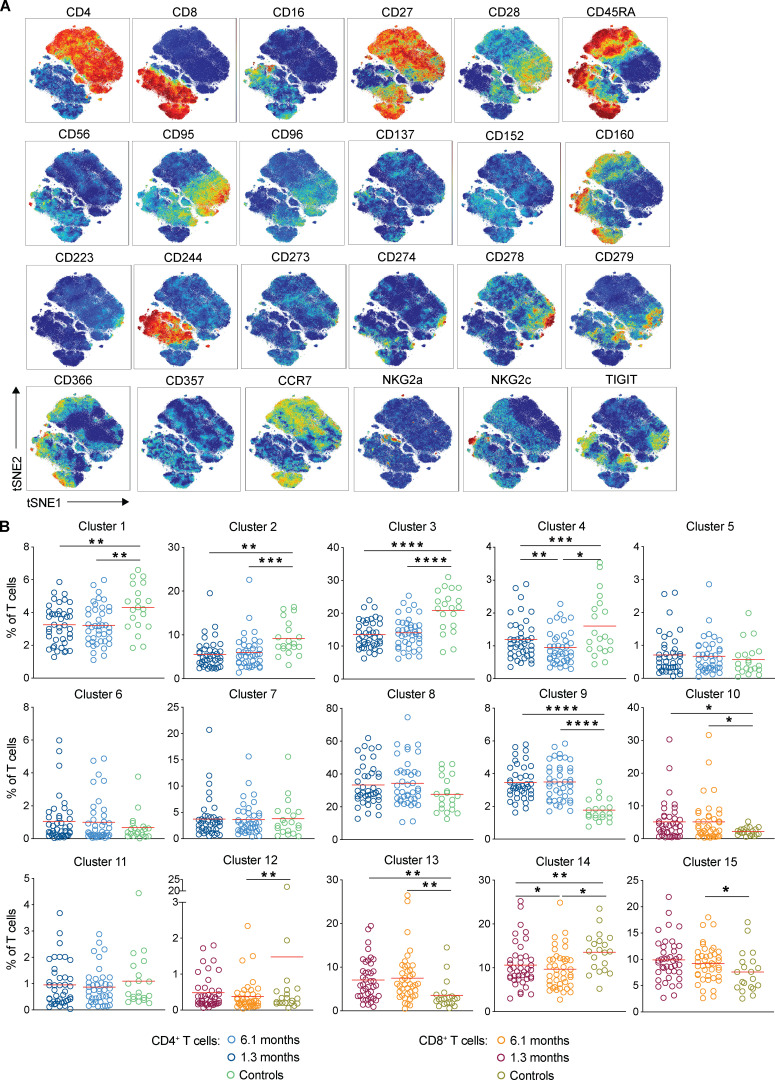
**Persistent longitudinal changes in the phenotypic landscape of T cells in individuals recovered from COVID-19, related to ****Fig. 1****.** **(A)** viSNE projections of the indicated protein expression. **(B)** Frequency of T cells from each group in FlowSOM clusters indicated. *, P < 0.05; **, P < 0.01; ***, P < 0.001; ****, P < 0.0001.

The phenotypic landscape of circulating T cells was determined by high-dimensional flow cytometry at both time points and compared with pre–COVID-19 samples from healthy individuals (*n* = 20; Table S2). Global high-dimensional mapping with t-distributed stochastic neighbor embedding (tSNE) revealed significant persistent alterations in SARS-CoV-2–recovered individuals (Fig. 1 A). Relatively underrepresented T cell clusters in recovered individuals included clusters 1, 2, 3, 4, and 14 (Fig. 1, B–D; and Fig. S1 B). Clusters 1, 3, and 4 showed a phenotype consistent with CD4^+^ central memory T cells (T_CM_ cells; CD45RA^−^CD27^+^CCR7^+^; Fig. 1 B, and C; and Fig. S1 A). In contrast, cluster 14 displayed features of CD8^+^ T_CM_ (CD45RA^−^CD27^+^CCR7^+^; Fig. 1, B and C; and Fig. S1 A). Examples of clusters showing a relative increase over control included clusters 10 and 13 (Fig. 1, B–D; and Fig. S1 B). Cluster 10 resembles CD8^+^ T effector cells (T_E_ cells; CD45RA^+^CD27^−^CCR7^−^, Fig. 1, B and C; and Fig. S1 A) and cluster 13 CD8^+^ effector memory T cells (T_EM_ cells; CD45RA^−^CD27^−^CCR7^−^; Fig. 1, B and C; and Fig. S1 A). The relative distribution of all of the clusters described above remained significantly different from control at the 6.1-mo time point (Fig. 1 D and Fig. S1 B). We conclude that there are significant shifts in circulating CD4^+^ and CD8^+^ memory T cell compartments that persist for half a year after SARS-CoV-2 infection.

**Figure S2.**
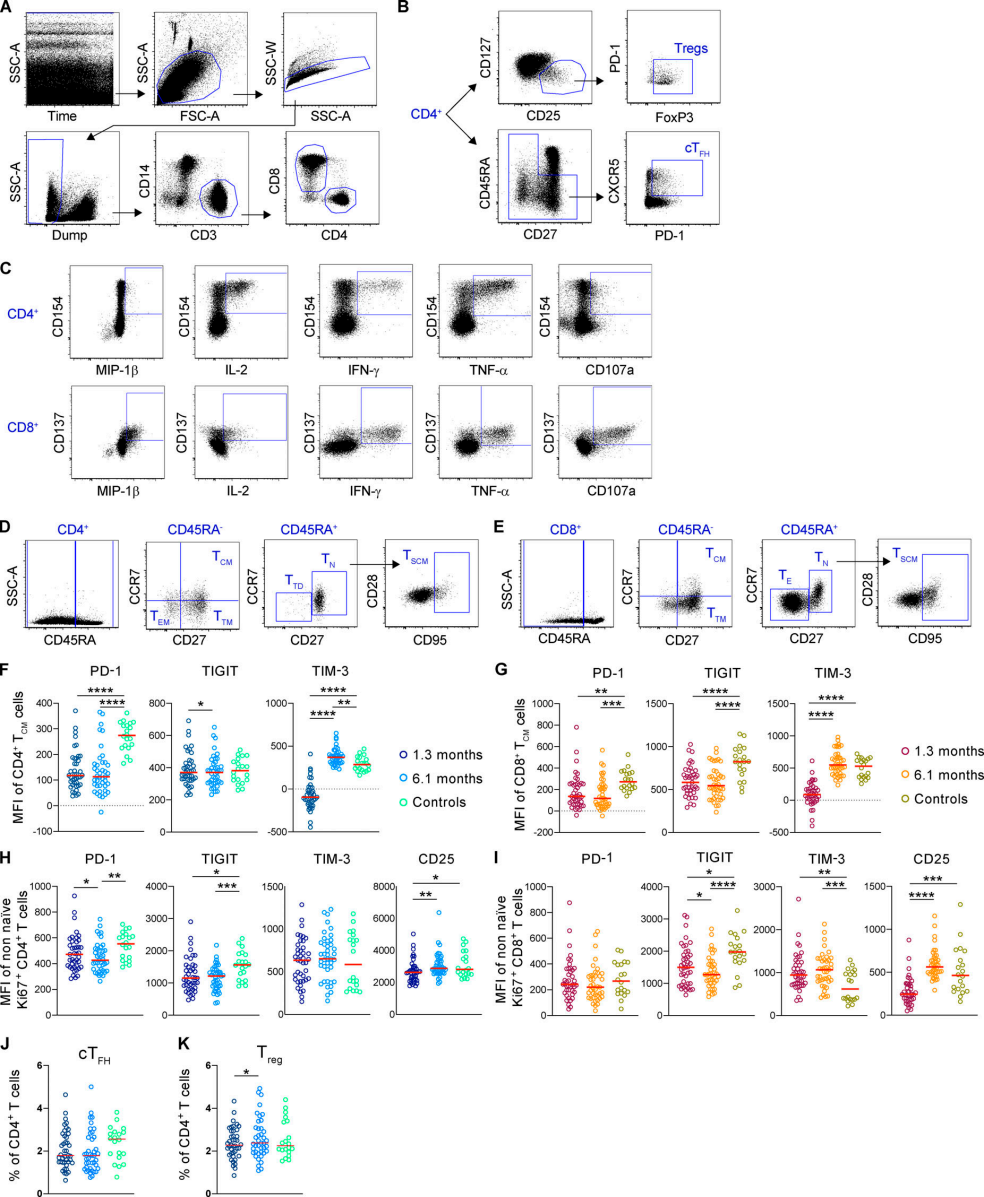
**Gating strategy and phenotyping of CD4^+^ and CD8^+^ T cells and their subsets in the peripheral blood of COVID-19 infected individuals.** **(A)** Gating strategy of total CD4^+^ and CD8^+^ T cells. Dump channel includes CD19, CD20, and CD66b as well as Live/Dead. CD4^+^ T cells are identified as Dump^−^CD14^−^CD3^+^CD4^+^, whereas CD8^+^ T cells are Dump^−^CD14^−^CD3^+^CD8^+^. **(B)** Gating strategy of cT_FH_ cells and T reg cells. cT_FH_ cells are gated as nonnaive CD4^+^ T cells that express PD-1 and CXCR5. T reg cells are CD4^+^CD25^+^CD127^dim^ FoxP3^+^ T cells. **(C)** Representative flow cytometry plots showing the cytokine production (MIP-1β, IL-2, IFN-γ, TNF-α, and CD107a) of CD4^+^ and CD8^+^ T cells after *Staphylococcus* enterotoxin B stimulation. **(D and E)** Flow cytometry gating strategy to identify CD4^+^ (D) and CD8^+^ (E) T cell subsets. T_SCM_ (stem cell memory), T_N_ (naive), T_CM_, T_TM_ (transitional memory), T_EM_, and T_TD_ (terminally differentiated)/T_E_ cell subsets are identified based on their CD45RA, CCR7, CD27, and CD95 expression. **(F)** PD-1, TIGIT, and TIM-3 expression of CD4^+^ central memory T cells. **(G)** PD-1, TIGIT, and TIM-3 expression of CD8^+^ central memory T cells. **(H)** PD-1, TIGIT, TIM-3, and CD25 expression of CD4^+^ cycling T cells (Ki67^+^). **(I)** PD-1, TIGIT, TIM-3, and CD25 expression of CD8^+^ cycling T cells (Ki67^+^). **(J and K)** cT_FH_ cell (J) and T reg cell (K) relative numbers. Each dot represents a COVID-19–convalescent individual (*n* = 41) at 1.3 mo (dark blue) or 6.1 mo (light blue) or control individuals (*n* = 20; green). Significance was determined by paired *t* test for comparisons between time points within individuals and unpaired *t* test for comparison between unexposed and COVID-19 individuals. *, P < 0.05; **, P < 0.01; ***, P < 0.001; ****, P < 0.0001. SSC-A, side scatter area; SSC-W, side scatter width; FSC-A, forward scatter area.

**Figure 2.**
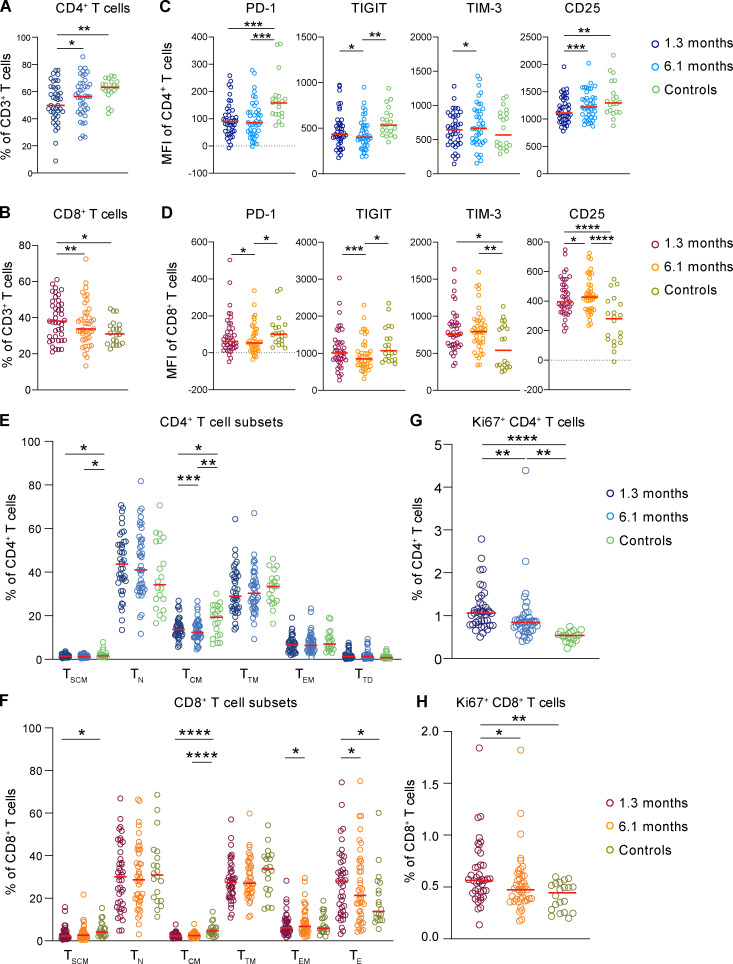
**Persistent changes after 6.1 mo.** **(A)** Frequency of CD4^+^ T cells out of total CD3^+^ T cells. **(B)** Frequency of CD8^+^ T cells out of total CD3^+^ T cells. **(C)** PD-1, TIGIT, TIM-3, and CD25 expression of CD4^+^ T cells. **(D)** PD-1, TIGIT, TIM-3, and CD25 expression of CD8^+^ T cells. **(E)** Percentage of T_SCM_ (stem cell memory), T_N_ (naive), T_CM _, T_TM_ (transitional memory), T_EM_, and T_TD_ (terminally differentiated) CD4^+^ T cells. **(F)** Percentage of T_SCM_, T_N_, T_CM_, T_TM_, T_EM_, and T_E_ CD8^+^ T cells. **(G)** Frequency of cycling Ki67^+^ CD4^+^ T cells. **(H)** Frequency of cycling Ki67^+^ CD8^+^ T cells. Each dot represents an individual with COVID-19 (*n* = 41) at 1.3 mo (dark blue for CD4^+^ T cells and dark red for CD8^+^ T cells) or 6.1 mo (light blue for CD4^+^ T cells and orange for CD8^+^ T cells) as well as control individuals (*n* = 20; green). Significance determined by paired *t* test for comparisons between time points within individuals and unpaired *t* test for comparison between controls and COVID-19 individuals. *, P < 0.05; **, P < 0.01; ***, P < 0.001; ****, P < 0.0001.

To further examine changes in the T cell compartment, we queried the data by traditional gating (Fig. S2, A–E). The relative proportions of circulating CD4^+^ T cells decreased significantly 1.3 mo after infection, whereas the circulating CD8^+^ T cells increased, but both returned to near-physiological levels by 6.1 mo (Fig. 2, A and B). PD-1 expression is modulated on activated and exhausted T cells and is associated with acute and prolonged changes in T cell function after viral infection in mice (Schönrich and Raftery, 2019; Jubel et al., 2020). PD-1 expression was decreased on CD4^+^ and CD8^+^ T cells at both time points (Fig. 2, C and D). Consistent with these alterations, T cell immunoreceptor with Ig and ITIM domains (TIGIT), TIM-3, and CD25 expression were also significantly different from control (Fig. 2, C and D). We conclude that there are persistent changes in the distribution of circulating CD4^+^ and CD8^+^ T cells and their expression of activation/exhaustion markers.

Consistent with the clustering analysis, central memory CD4^+^ and CD8^+^ T cells decreased, and this defect persisted throughout the observation period (Fig. 2, E and F). In addition, there was an increase in cycling CD4^+^ and CD8^+^ T cells (Ki67^+^) at both time points (Fig. 2, G and H). Both central memory and cycling cells also showed lower levels of PD-1 expression at both time points (Fig. S2, F–I). In contrast, we found no significant changes in circulating T follicular helper (cT_FH_) cells or regulatory T cells (T reg cells; Fig. S2, J and K). Thus, the more traditional gating strategy is generally consistent with the tSNE analysis.

### SARS-CoV-2 antigen–specific CD4^+^ T cells

**Figure 3.**
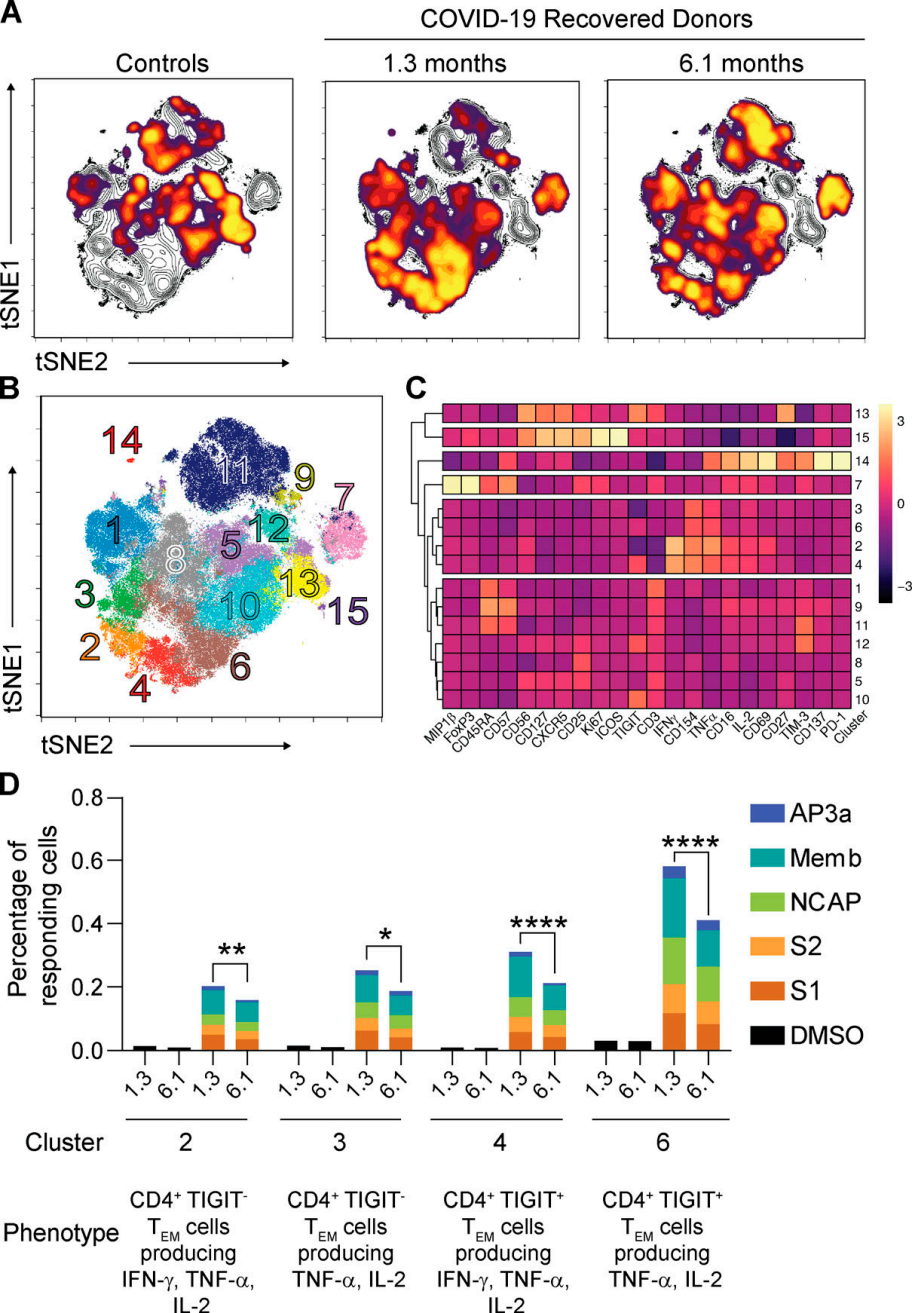
**Antigen-specific CD4^+^ T cells dynamics in COVID-19–convalescent individuals.** **(A)** viSNE representations of CD137^+^ CD154^+^ SARS-CoV-2-stimulated CD4^+^ T cells in unexposed individuals (controls, *n* = 20) and COVID-19 convalescent individuals (*n* = 41) pooled. Density plots from each group concatenated is overlaid on the total contour viSNE plot. **(B)** viSNE representation of antigen-specific CD4^+^ T cell clusters, identified by FlowSOM clustering. **(C)** Column-scaled *z*-scores of MFI as indicated by cluster and marker. **(D)** Percentage of antigen-specific CD4^+^ cells in the indicated FlowSOM clusters. Each bar represents the mean percentage for all COVID-19–convalescent individuals for the indicated SARS-CoV-2 peptide pools. *, P < 0.05; **, P < 0.01; ****, P < 0.0001.

**Figure S3.**
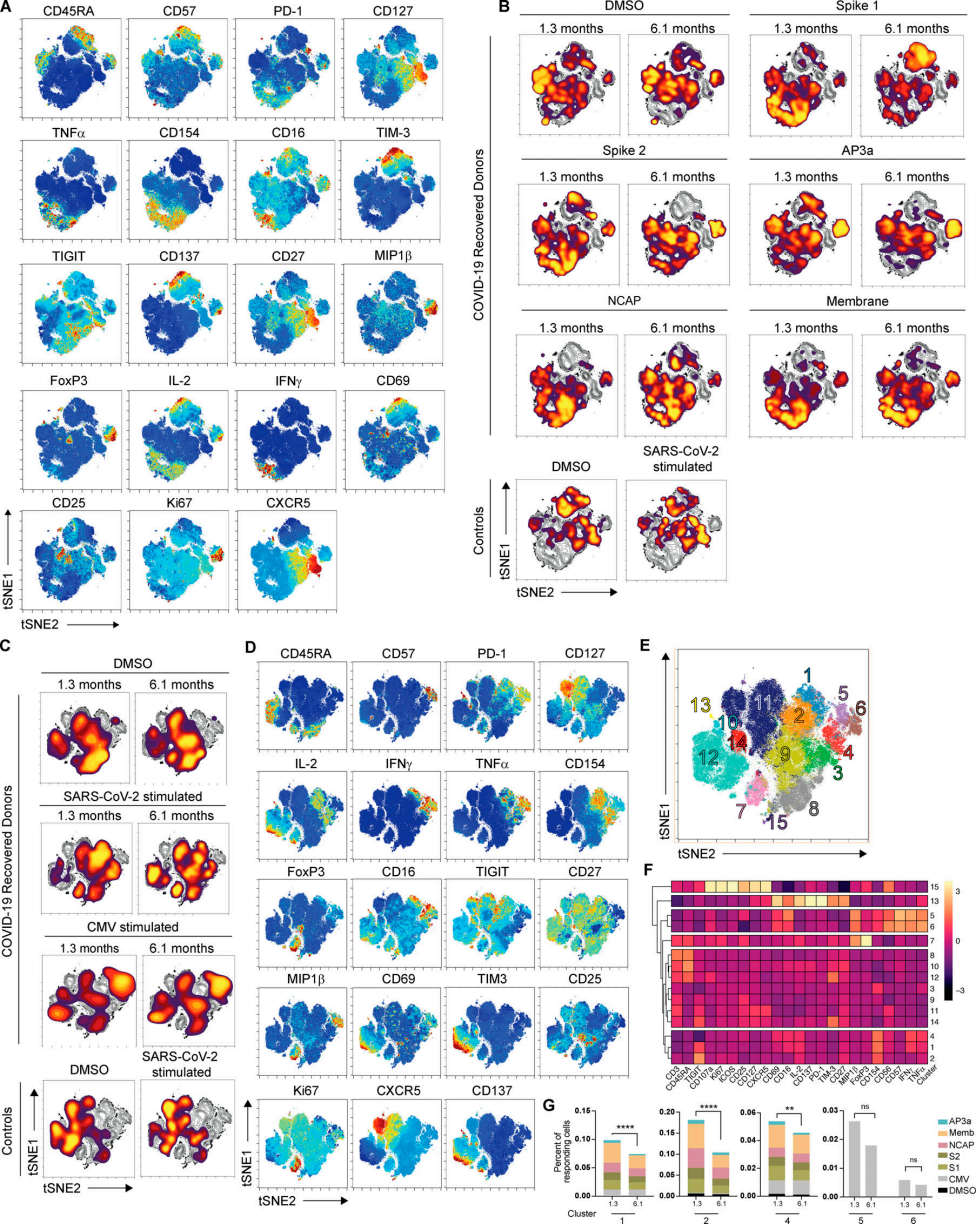
**Antigen-specific CD4^+^ T cell dynamics responding to individual SARS-CoV-2 peptide pools and CMV in COVID-19–convalescent individuals.** **(A)** viSNE projections of CD137^+^CD154^+^ SARS-CoV-2–stimulated CD4^+^ T cells in controls (*n* = 20) and COVID-19–convalescent individuals (*n* = 41) pooled, related to Fig. 3, with the indicated protein expression shown. **(B)** viSNE representations of CD137^+^CD154^+^ SARS-CoV-2–stimulated CD4^+^ T cells in COVID-19–convalescent individuals and controls pooled, related to Fig. 3. Density plots from COVID-19–recovered donors show responses to the DMSO control and to each individual SARS-CoV-2 peptide pool; and plots from controls show responses to the DMSO control and all SARS-CoV-2 peptide pools combined. Density plots are overlaid on the total contour viSNE plot. **(C)** viSNE representations of CD137^+^CD154^+^CD4^+^ T cells, including samples from stimulations with SARS-CoV-2 or CMV in controls (*n* = 20) and COVID-19–convalescent individuals (*n* = 41) pooled. Density plots from each group concatenated are overlaid on the total contour viSNE plot. **(D)** viSNE representation of each indicated marker expression, related to Fig. S3 C. **(E)** viSNE representation of CD137^+^CD154^+^CD4^+^ T cell clusters, identified by FlowSOM clustering, related to Fig. S3 C. **(F)** MFI for indicated markers, column-normalized z-score, related to Fig. S3 C. **(G)** Percentage of CD137^+^CD154^+^CD4^+^ cells in the indicated FlowSOM clusters. Each bar represents the mean percentage for all COVID-19 convalescent individuals for the indicated SARS-CoV-2 peptide pools or CMV, related to Fig. S3 C. *, P < 0.05; **, P < 0.01; ***, P < 0.001; ****, P < 0.0001; ns, not significant.

To investigate SARS-CoV-2 antigen–specific CD4^+^ T cells, we stimulated the cells with a collection of pooled SARS-CoV-2 peptides in vitro. COVID-19–convalescent individuals were compared with healthy donors by tSNE using high-dimensional flow cytometry. Antigen-specific CD4^+^ T cells expressing memory markers as well as IL-2, IFN-γ, TNF-α, and CD154 were markedly increased in COVID-19–recovered individuals compared with healthy donors, but the relative frequency of these cells decreased at the 6.1-mo time point (clusters 2, 3, 4, and 6; Fig. 3, A–D; and Fig. S3 A). The magnitude of the decrease in these responses between the time points varied between 22% and 32% depending on the cluster (Fig. 3 D) and the individual peptide pool (Fig. S3 B). In contrast, responses to cytomegalovirus (CMV) peptides remained unchanged between the two time points (Fig. S3, C–G). We conclude that SARS-CoV-2 antigen–specific CD4^+^ T cells are induced during acute infection and that they remain detectable after 6.1 mo.

**Figure 4.**
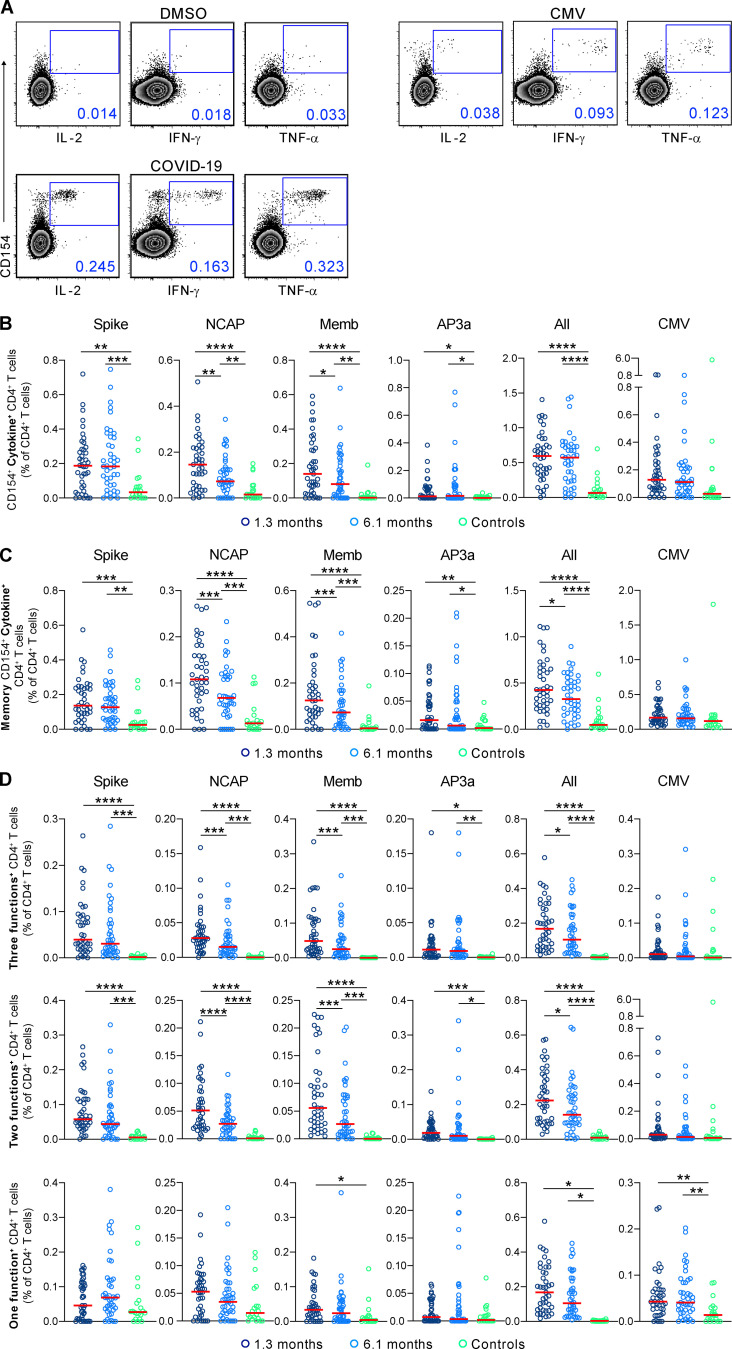
**SARS-CoV-2–specific CD4^+^ T cells responses in COVID-19–convalescent individuals.** Longitudinal analysis of COVID-19–specific CD4^+^ T cell responses in paired samples obtained 1.3 mo and 6.1 mo after infection. Cytokine production (IL-2, IFN-γ, and TNF-α) by CD4^+^ T cells analyzed by intracellular cytokine staining. Spike (aggregation of responses to Spike peptide pool S1 and S2), NCAP, Memb, and nonstructural AP3a peptide pools responses by controls (*n* = 20) and COVID-19–convalescent individuals (*n* = 41). **(A)** Gating strategy for identification of SARS-CoV-2–specific CD4^+^ T cells. **(B)** Combined frequency of SARS-CoV-2–specific CD4^+^ T cells that produce IL-2, IFN-γ, and/or TNF-α. **(C)** Frequency of SARS-CoV-2–specific memory CD4^+^ T cells (CD45RA^−^CD27^+^) that produce IL-2, IFN-γ, and/or TNF-α. **(D)** Frequency of SARS-CoV-2–specific CD4^+^ T cells that produce three cytokines, two cytokines, or one cytokine. Each dot represents an individual with COVID-19 (*n* = 41) at 1.3 mo (dark blue) or 6.1 mo (light blue) or control individuals (*n* = 20; green). Significance was determined by paired *t* test for comparisons between time points within individuals and unpaired *t* test for comparison between controls and COVID-19 individuals. *, P < 0.05; **, P < 0.01; ***, P < 0.001; ****, P < 0.0001.

**Figure S4.**
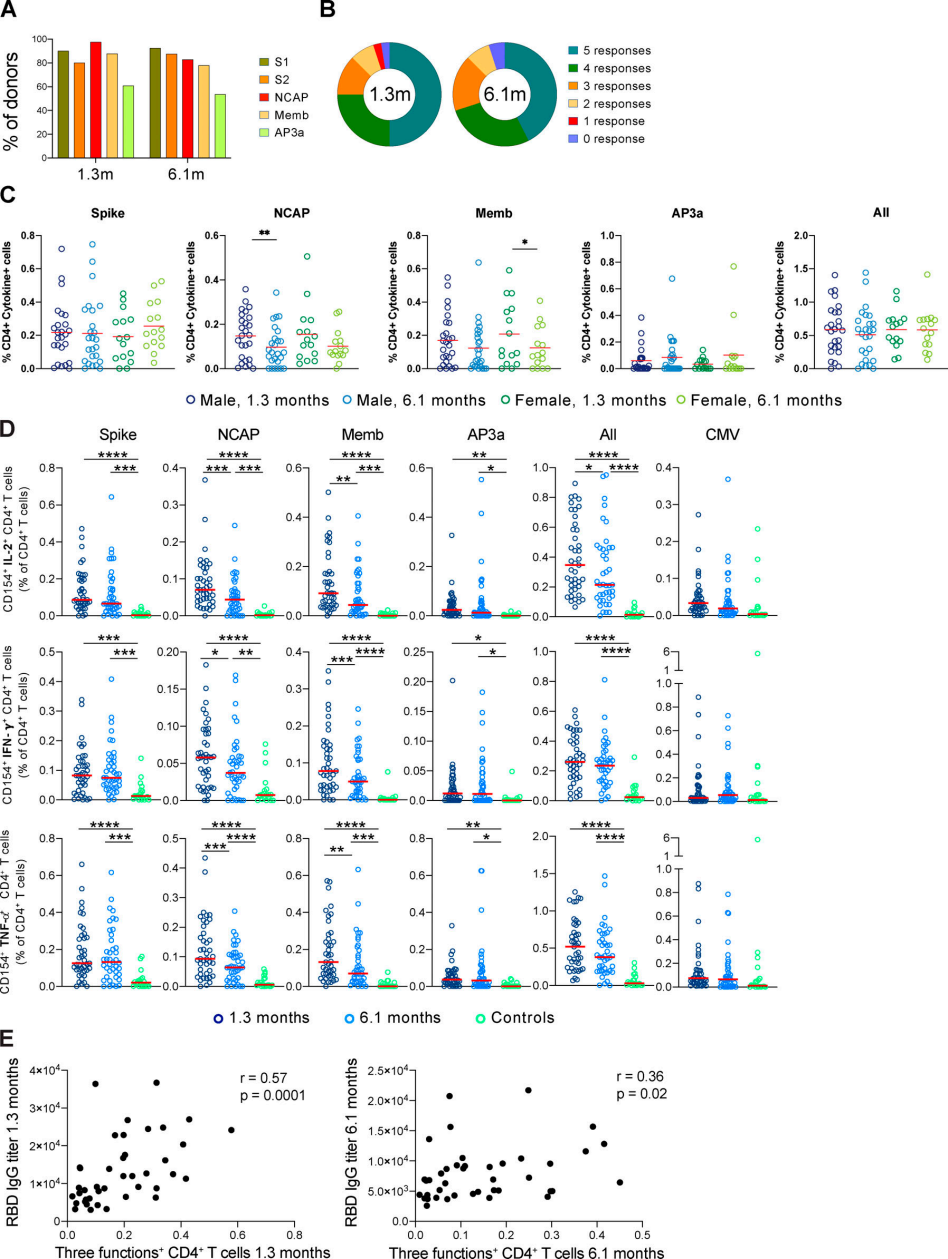
**SARS-CoV-2–specific CD4^+^ T cell responses in COVID-19–convalescent individuals, related to ****Fig. 4****.** **(A)** Percentage of COVID-19 individuals (*n* = 41) who respond to Spike (responses to Spike peptide pool S1 and S2), NCAP, Memb, and nonstructural AP3a peptide pools at 1.3 or 6.1 mo. **(B)** Pie chart shows the frequency of recovered COVID-19 individuals (*n* = 41) who respond to one, two, three, four, or five peptide pools. **(C)** Frequency of cytokine-expressing cells among CD4^+^ T cells following stimulation with indicated peptide and all frequencies pooled, for indicated sex and time point. **(D)** Frequency of SARS-CoV-2–specific CD4^+^ T cells that produce IL-2, IFN-γ, or TNF-α. Each dot represents an individual with COVID-19 at 1.3 mo (dark blue) or 6.1 mo (light blue) or control individuals (green). **(E)** Normalized area under the curve for IgG anti-RBD plotted against the relative frequency of CD4^+^ T cells producing three cytokines (three functions). The r and P values were determined by two-tailed Spearman’s correlations. Significance (C and D) was determined by paired *t* test for comparisons between time points within individuals and unpaired *t* test for comparison between controls (*n* = 20) and COVID-19 individuals (*n* = 41). *, P < 0.05; **, P < 0.01; ***, P < 0.001; ****, P < 0.0001.

To characterize SARS-CoV-2 antigen–specific cytokine-producing CD4^+^ T cells, we analyzed the high-dimensional flow cytometry data by traditional gating (Fig. 4 and Fig. S2, A and C). IL-2, INF-γ, and TNF-α responses to peptide pools corresponding to Spike (S1 and S2), nucleocapsid protein (NCAP), membrane protein (Memb), AP3a, and CMV control were measured independently at both time points (Fig. 4, A–D). In all cases, responses to the individual peptide pools were elevated above control at both time points, and all but NCAP and Memb responses remained stable between the time points (Fig. 4 B). Among the individuals tested, 97.5% and 95% responded to at least one of the antigens at 1.3 and 6.1 mo, respectively (Fig. S4, A and B). When all cytokines are considered together, we find a significant increase in antigen-specific CD4^+^ T cell responses to all of the individual peptide pools at both time points compared with control, with few differences between male and females (Fig. 4 B and Fig. S4 C). The increase in CD4^+^ T cell cytokine responses to the individual peptide pools and the overall combination of all SARS-CoV-2 antigens was not driven by any single cytokine but instead reflected increases in each of the three cytokines measured (Fig. S4 D). Moreover, when all antigen-specific responses are considered in aggregate, the fraction of responding CD4^+^ T cells remains significantly elevated and is not different between the two time points (Fig. 4 B). Antigen-specific CD4^+^ memory T cells (CD45RA^−^CD27^+^) were similarly elevated at the two time points (Fig. 4 C). Finally, there was no significant change in the CD4^+^ T cell response by the same individuals to the control CMV peptide pool (Fig. 4, B–D).

Polyfunctional cytokine responses are associated with effective cellular immune responses (Seder et al., 2008; Lin et al., 2015; Betts et al., 2006). Polyfunctional CD4^+^ T cells responses to each of the individual peptide pools were significantly elevated at the early time point and remained so after 6.1 mo (Fig. 4 D). The magnitude of these responses was directly correlated with antibodies to the SARS-CoV-2 receptor binding domain (Fig. S4 E). However, there was a decrease in polyfunctional CD4^+^ T cell responses to NCAP and Memb antigens at 6.1 mo, which is also reflected in a 22% decrease in the overall trifunctional response to the combined SARS-CoV-2 peptide libraries (Fig. 4 D). In contrast, CD4^+^ T cells that produced only a single cytokine were only elevated in response to Memb and only at the early time point (Fig. 4 D). We conclude that robust polyfunctional CD4^+^ T cell responses persist for 6.1 mo after SARS-CoV-2 infection but decrease significantly when compared with an earlier time point.

### SARS-CoV-2 antigen–specific CD8^+^ T cells

**Figure 5.**
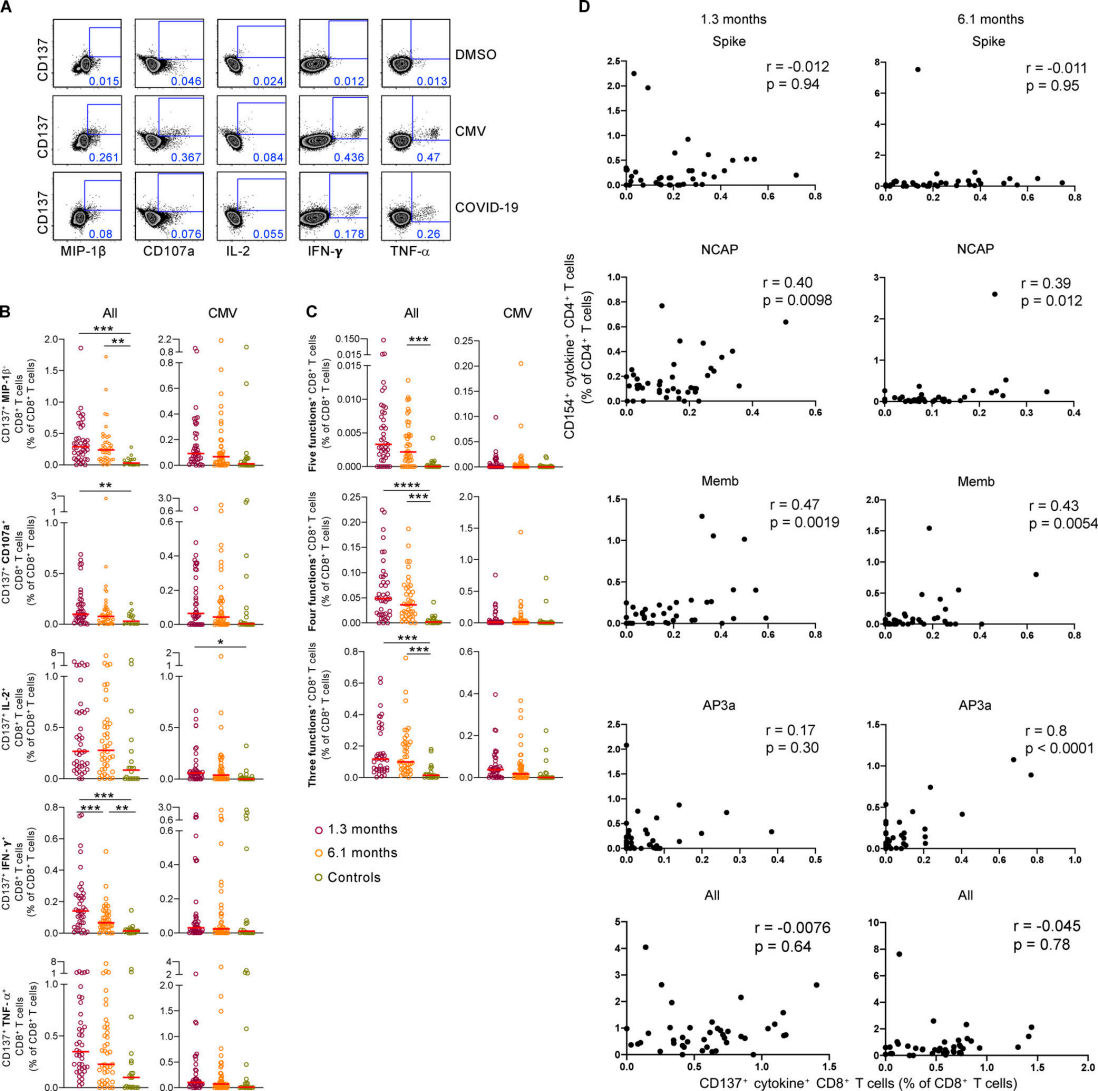
**SARS-CoV-2–specific CD8^+^ T cells responses in COVID-19–convalescent individuals.** Longitudinal analysis of COVID-19–specific CD8^+^ T cell responses in paired samples obtained from the same individual at 1.3 and 6.1 mo after infection. Cytokine production (MIP-1β, CD107a, IL-2, IFN-γ, and TNF-α) by CD8^+^ T cells analyzed by intracellular cytokine staining. Spike (aggregation of responses to Spike peptide pool S1 and S2), NCAP, Memb, and nonstructural AP3a peptide pools responses by controls (*n* = 20) and convalescent COVID-19 individuals (*n* = 41). **(A)** Gating strategy for identification of SARS-CoV-2–specific CD8^+^ T cells. **(B)** Frequency of SARS-CoV-2–specific CD8^+^ T cells that produce MIP-1β, CD107a, IL-2, IFN-γ, or TNF-α. **(C)** Frequency of SARS-CoV-2–specific CD8^+^ T cells that produce five cytokines, four cytokines, or three cytokines. Each dot represents an individual with COVID-19 (*n* = 41) at 1.3 mo (dark red) or 6.1 mo (orange) or control individuals (*n* = 20; green). Significance determined by paired *t* test for comparisons between time points within individuals and unpaired *t* test for comparison between controls and COVID-19 individuals. *, P < 0.05; **, P < 0.01; ***, P < 0.001; ****, P < 0.0001. **(D)** Frequency of CD154^+^ cytokine^+^ CD4^+^ T cells versus frequency of CD137^+^ cytokine^+^ CD8^+^ T cells after stimulation with indicated peptide. The r and P values were determined by two-tailed Spearman correlations.

**Figure S5.**
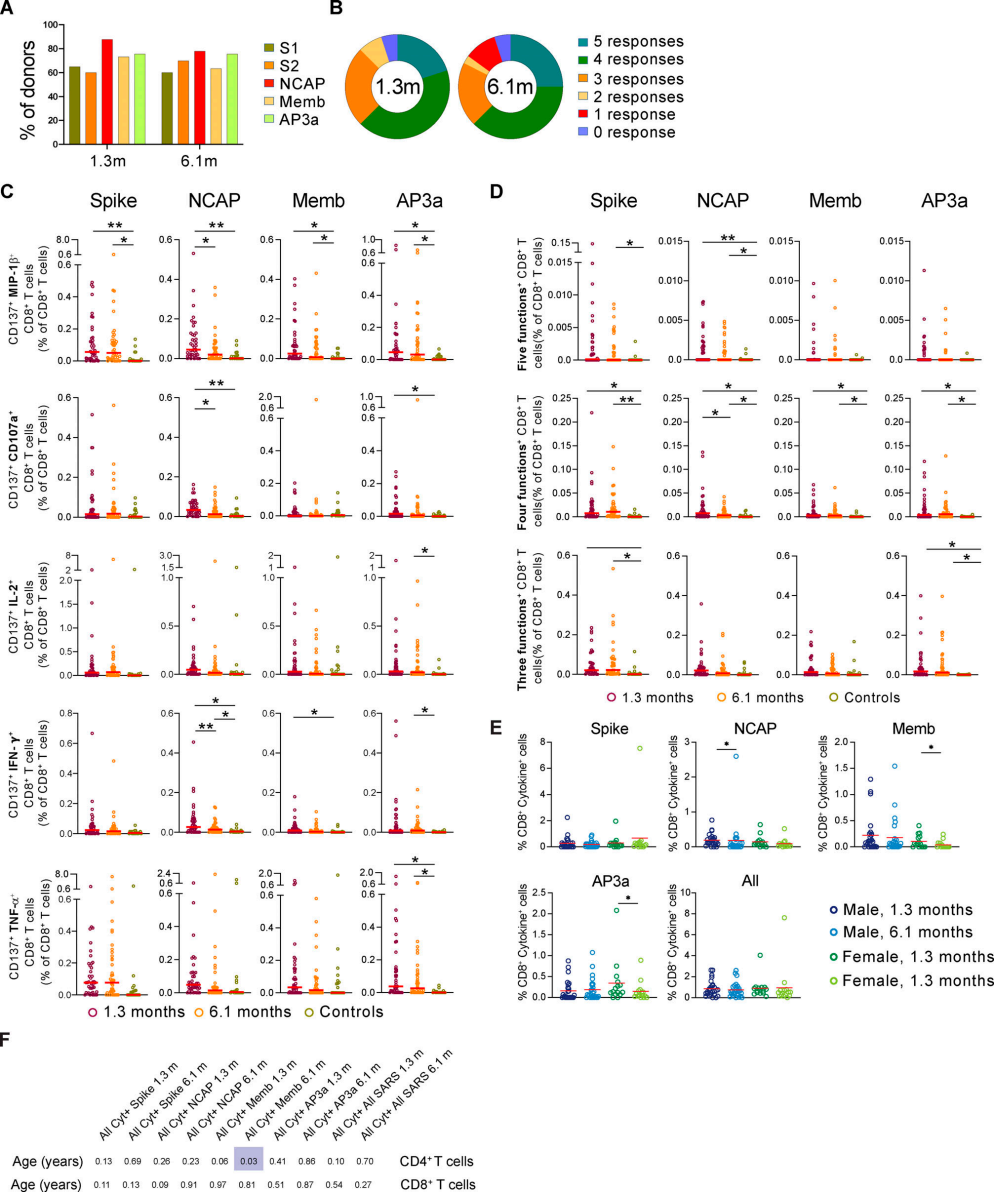
**SARS-CoV-2–specific CD8^+^ T cell responses in COVID-19–convalescent individuals, related to ****Fig. 5****.** **(A)** Percentage of COVID-19 individuals (*n* = 41) who respond to Spike (responses to Spike peptide pool S1 and S2), NCAP, Memb, and nonstructural AP3 peptide pools at 1.3 or 6.1 mo. **(B)** Pie chart shows the frequency of mild COVID-19 individuals who have CD8^+^ responses to one, two, three, four, or five peptide pools. **(C)** Frequency of SARS-CoV-2–specific CD8^+^ T cells that produce either MIP-1β, CD107a, IL-2, IFN-γ, or TNF-α. **(D)** Frequency of SARS-CoV-2–specific CD8^+^ T cells that produce five, four, or three cytokines. Each dot represents an individual with COVID-19 at 1.3 mo (dark red) or 6.1 mo (orange) or unexposed individuals (green). **(E)** Frequency of cytokine-expressing cells among CD8^+^ T cells following stimulation with indicated peptide and all frequencies pooled, for indicated sex and time point. **(F)** P values for correlations between age and antigen-specific CD4^+^ and CD8^+^ T cells. Significance was determined by paired *t* test for comparisons between time points within individuals and unpaired *t* test for comparison between controls (*n* = 20) and COVID-19 individuals (*n* = 41). *, P < 0.05; **, P < 0.01.

To examine antigen-specific CD8^+^ T cell responses, we measured production of Mip-1β, CD107a, IL-2, INF-γ, and TNF-α in response to stimulation with SARS-CoV-2 peptide pools in vitro. In contrast to CD4^+^ T cells, CD8^+^ T cell responses were far more variable and generally less robust, making tSNE analysis less reliable, and therefore, these responses were only analyzed by traditional gating (Fig. 5 and Fig. S2, A and C). Although 95% of the donors tested responded to at least one of the peptide pools at both 1.3 and 6.1 mo, the percentage of responding cells was low (Fig. 5 A and Fig. S5, A–D). When all peptide responses were pooled, we found significant Mip-1β and INF-γ responses at both time points, while CD107a was only increased above control at 1.3 mo (Fig. 5 B). Polyfunctional responses that included at least three different cytokines were also elevated at both time points (Fig. 5 C). Analysis of CD8^+^ T cell responses to the individual peptide pools when all cytokines are considered together revealed no significant differences in the frequency of these subsets over time between sexes (with an exception for NCAP, which is slightly decreased in males, and Memb and AP3a in females; Fig. S5 E). Additionally, our data do not demonstrate a strong association between SARS-CoV-2–specific T-helper responses and cytotoxic responses when all responses are aggregated (Fig. 5 D). Furthermore, antigen-specific CD4^+^ and CD8^+^ T cells did not show any consistent correlation with age (Fig. S5 F). Overall, we conclude that although we detect fewer antigen-specific CD8^+^ than CD4^+^ T cells to SARS-CoV-2, they persist 6.1 mo after infection.

## Discussion

Most effective vaccines and anamnestic responses to pathogens are mediated by neutralizing antibodies (Plotkin, 2020; Inoue et al., 2018; Weisel and Shlomchik, 2017). Consistent with this notion, neutralizing antibodies are protective against SARS-CoV-2 infection in animal models (Deng et al., 2020) and appear to correlate with protection in vaccinated humans (Gaebler and Nussenzweig, 2020). These responses are dependent on specialized helper T cells that control the activation and selection of antibody producing plasma and memory B cells (Victora and Nussenzweig, 2012; Crotty, 2015).

CD4^+^ and CD8^+^ T cells can also contribute directly to protection against SARS-CoV (Le Bert et al., 2020; Tang et al., 2011; Yang et al., 2006; Channappanavar et al., 2014) and other viral pathogens in animal models (Schmitz et al., 1999; Shoukry et al., 2003; Snyder, 2011). In addition, control of human viral pathogens such as HIV-1 is associated with CD8^+^ T cells in rare elite controllers (Collins et al., 2020). To gain further understanding into whether SARS-CoV-2 infection is associated with enduring T cell responses, we examined cellular immunity to SARS-CoV-2 in paired samples collected 1.3 and 6.1 mo after infection.

We find persistent changes in the overall CD4^+^ and CD8^+^ T cell compartments irrespective of the ability of the cells in these compartments to react to antigen. For example, the relative number of CD4^+^ and CD8^+^ central memory T cells decreases in infected individuals at 1.3 mo and remains so even after 6 mo when compared with healthy controls. In contrast, polyfunctional SARS-CoV-2 antigen–specific CD4^+^ and CD8^+^ memory T cells were elevated at the same time points. The persistence of antigen-specific memory T cells is directly correlated with and parallels the B cell compartment (Rodda et al., 2021; Dan et al., 2020 *Preprint*; Gaebler et al., 2020* Preprint*). Despite decreasing serum levels of anti–SARS-CoV-2 neutralizing antibodies, the memory B cell compartment continues to evolve over the first 6 mo after infection, including increased levels of somatic mutations and increasing resistance to escape mutations in the receptor-binding domain (RBD; Gaebler et al., 2020). Continuing antibody evolution and persistent alterations in the memory T cell compartment may be due to residual antigen in germinal centers (Victora and Nussenzweig, 2012) and cellular compartments rich in ACE-2 expressing cells like the brush border epithelial cells in the gut (Gaebler et al., 2020* Preprint*). Similar to SARS-CoV and Middle East respiratory syndrome infections, SARS-CoV-2 antigen–specific memory CD4^+^ and CD8^+^ T and B cells persist and would be expected to play role in protection against reexposure.

In addition to persistent antigen-specific memory responses, chronic viral infections such as HIV are associated with lasting immune perturbations (Breton et al., 2013), but little is known about acute viral infections. SARS-CoV-2 is an acute infection that typically resolves after 2–3 wk and in rare instances leads to severe lung disease and mortality (Richardson et al., 2020; O'Driscoll et al., 2020). The cohort we examined is biased toward males (63.4%) and Caucasians with milder forms of the disease; nevertheless, CD4^+^ and CD8^+^ memory T cell subset distribution, cell division, and expression of activation/exhaustion markers remain altered 6 mo compared with control after SARS-CoV-2 infection. These persistent differences were not directly associated with persistent symptoms, and their impact on the overall immune health of the individual remains to be determined.

## Materials and methods

### Study participants

Study participants (*n* = 61) were residents of the greater New York City tristate region, 20 of whom were SARS-CoV-2 unexposed (prepandemic; median age, 52.5 yr; 45% female; Table S2), and 41 were SARS-CoV-2 infected (median age, 45 yr; 36.6% female; Table S1). Previously enrolled study participants (Robbiani et al., 2020) were asked to return for a 6-mo follow up visit at the Rockefeller University Hospital in New York from August 31 through October 16, 2020. All of the individuals tested had RT-PCR–confirmed SARS-CoV-2 infection or were close contacts who seroconverted. A summary of the participants’ clinical characteristics is presented in Table S1 and has been extensively described elsewhere (Robbiani et al., 2020; Gaebler et al., 2020* Preprint*). All participants provided written informed consent before participation in the study, and the study was conducted in accordance with Good Clinical Practice and clinical data collection. The study was performed in compliance with all relevant ethical regulations, and the protocol was approved by the Institutional Review Board of The Rockefeller University.

### Cell preparation

Blood samples were collected an average of 1.3 and 6.1 mo after infection (Table S1). Peripheral blood mononuclear cells (PBMCs) were isolated from heparinized blood by density gradient centrifugation (Ficoll-Paque) and cryopreserved in 90% heat-activated FBS plus 10% DMSO in liquid nitrogen. Thawed PBMCs were washed and resuspended at 2 × 10^6^ cells/ml with RPMI 1640 supplemented with 10% heat-inactivated human serum (GemCell) and 10 U/ml Benzonase. For cell stimulation experiments, cells were rested at 37°C and 5% CO_2_ for 8 h before stimulation with peptides for use in intracellular cytokine staining assays.

### Synthetic COVID-19 peptides

We purchased from JPT the following SARS-CoV-2 peptide pools: pool of 315 peptides covering the spike (15mers with 11-aa overlap; delivered in two subpools of 158 [S1 pool] and 157 [S2 pool] peptides), pool of 102 peptides covering the nucleoprotein, pool of 53 peptides covering the membrane, and pool of 66 peptides covering AP3a. A pool of 138 peptides (15mers with 11-aa overlap) derived from 65-kD phosphoprotein (pp65) of human CMV (JPT) was used as a control. *Staphylococcus* enterotoxin B (Sigma-Aldrich) was used at a final concentration of 1 µg/ml as a positive stimulation control. Peptides were reconstituted in high-grade DMSO (Sigma-Aldrich) at a concentration of 0.1 mg/ml and used at a final concentration of 0.25 µg/ml in a maximum of 0.2% DMSO. PBMCs with peptide diluent (0.2% DMSO) served as the negative control.

### Cell stimulation and intracellular staining

Cells were stimulated for 12 h with SARS-CoV-2 peptide pools (0.25 µg/ml) in the presence of αCD28/αCD49d costimulatory antibodies (BD FastImmune; BD Biosciences), 5 µg/ml brefeldin A (Sigma-Aldrich), 5 µg/ml Monensin. (BD GolgiStop; BD Biosciences), and anti-CD107a-PE-Cy5 (Clone H4A3; the staining for CD107a is performed during cell activation in this assay). A negative control containing PBMCs and costimulatory antibodies from the same subject, with DMSO, was also included for each assay. Following stimulation, cells were washed with PBS and first stained with CCR7 (Clone 3D12) at 37°C for 20 min in PBS containing 2% FBS. Cells were then surface stained for 30 min in the dark at 4°C with viability reagent (BD Horizon Fixable Viability Stain; BD Biosciences) and a 29-color cocktail of mAbs containing surface antibodies against CD19 (Clone SJ25C1), CD20 (Clone 2H7), CD66b (Clone G10F5), CD14 (Clone M5E2), CD16 (Clone 3G8), CD56 (Clone B159), CD4 (Clone OKT4), CD8 (Clone RPA-T8), CD45RA (Clone HI100), CD27 (Clone M-T271), CD57 (Clone NK-1), CD25 (Clone 2A3), CXCR5 (Clone RF8B2), CD127 (Clone HIL-7R-M21), PD-1 (Clone EH12.1), TIGIT (Clone 741182), TIM-3 (Clone 7D3), and CD69 (Clone FN50). The cells were then washed with PBS containing 2% FBS and permeabilized according to the manufacturer’s instructions using a Foxp3/Transcription Factor Staining Buffer Set (eBioscience) and stained with intracellular antibodies against CD3 (Clone SK7), MIP-1b (Clone D21-1351), IL-2 (Clone MQ1-17H12), IFN-γ (Clone B27), TNF-α (Clone MAb11), FoxP3 (Clone 236A/E7), Ki67 (Clone ki-67), CD154 (Clone 24–3), and CD137 (Clone 4B4-1). After labeling, cells were washed and fixed in PBS containing 2% paraformaldehyde and stored at 4°C before flow cytometry acquisition within 24 h.

### Phenotypic characterization and surface staining

Cells from all donors were initially stained with CCR7 (Clone 3D12) at 37°C for 20 min in PBS containing 2% FBS. Cells were then stained with a 29-color staining cocktail containing viability reagent and surface antibodies against CD19 (Clone SJ25C1), CD20 (Clone 2H7), CD66b (Clone G10F5), CD14 (Clone M5E2), CD16 (Clone 3G8), CD56 (Clone B159), CD3 (Clone SK7), CD4 (Clone OKT4), CD8 (Clone RPA-T8), CD45RA (Clone HI100), CD27 (Clone M-T271), CD95 (Clone DX2), NKG2A (Clone 131411), NKG2C (Clone 134591), PD-1 (Clone EH12.1), TIGIT (Clone 741182), TIM-3 (Clone 7D3), CD152 (Clone BNI3), CD272 (Clone J168-540), CD134 (Clone ACT35), CD274 (Clone MIH1), CD273 (Clone MIH18), CD96 (Clone 6F9), CD357 (Clone V27-580), CD160 (Clone BY55), CD137 (Clone 4B4-1), CD223 (Clone T47-530), CD278 (Clone C398.4A), CD28 (Clone CD28.2), and CD244 (Clone eBioC1.7). Titrated antibodies were added to 2 million cells in 50 µl PBS containing 2% FBS for 30 min at 4°C. Washed cells were then fixed in 2% formaldehyde and stored at 4°C until analysis.

### Flow cytometry analysis

All events (∼1,200,000–1,800,000 events per sample) were collected on a BD FACSymphony A5 Cell Analyzer (BD Biosciences). The lymphocytes were gated for further analysis, as described in Fig. S2, A–E, using FlowJo version 9.9.6 (BD Biosciences). For cytokine expression, we subtracted the background in the negative control. To ensure equivalent fluorescence intensities (median fluorescence intensity [MFI]) from one experiment to another, we used Rainbow beads (Spherotech). For every run, Rainbow beads were acquired first and the voltages were adjusted if necessary to accommodate both daily variations and larger changes in performance such as would be seen after cytometer maintenance and alignment.

### High-dimensional data analysis of flow cytometry data

viSNE and FlowSOM analyses were performed on Cytobank (https://cytobank.org). viSNE analysis was performed using equal sampling of 5,000 cells (for total T cell phenotyping analysis; Fig. 1) or proportional sampling (for antigen-specific CD4^+^ T cell analysis; Fig. 3) from each FCS file, with 7,500 iterations, a perplexity of 30, and a theta of 0.5. The following markers were used to generate viSNE maps: CD273, CD96, NKG2C, CD152, TIGIT, CD272, CD134, CD45RA, CD137, CD366, CD95, CD279, CD16, CD274, CD27, CD56, CD357, CCR7, CD223, CD278, CD28, CD244, NKG2a, CD4, and CD8 for total T cells (Fig. 1 and Fig. S1, A and B) and CXCR5, CD127, MIP1β, CD137, IL-2, TIGIT, CD45RA, CD57, CD25, TIM-3, CD69, PD-1, CD16, IFN-γ, CD27, CD56, TNF-α, Ki67, ICOS, CD107a, FoxP3, and CD154 for antigen-specific CD4^+^ T cells (Fig. 3 and Fig. S3 A–G). Resulting viSNE maps were fed into the FlowSOM clustering algorithm (Van Gassen et al., 2015). The self-organizing map was generated using hierarchical consensus clustering on the tSNE axes.

### Statistical analysis

Statistical analyses were performed using Prism 8.0 (GraphPad). Significances between matched groups were calculated using paired *t* test, whereas differences between unmatched groups were compared using unpaired *t* test or one-way ANOVA. Correlations were performed using the Spearman rank correlation test.

### Online supplemental material

Fig. S1 comprehensively details the phenotypic landscape of circulating CD4^+^ and CD8^+^ T cells at 1.3 and 6.1 mo after infection from a cohort of 41 COVID-19–convalescent volunteers compared with pre–COVID-19 samples from healthy individuals. Fig. S2 shows the gating strategy used to define CD4^+^ and CD8^+^ T cell subsets in flow cytometry datasets as well as their frequencies and expression analysis. Fig. S3 comprehensively details the phenotypic landscape of SARS-CoV-2 and CMV antigen-specific CD4^+^ T cells at 1.3 and 6.1 mo after infection from a cohort of 41 COVID-19–convalescent volunteers compared with pre–COVID-19 samples from healthy individuals. Fig. S4 displays cytokine profiles of SARS-CoV-2 CD4^+^ T cell responses in COVID-19–convalescent volunteers and controls. Fig. S5 displays cytokine profiles of SARS-CoV-2 CD8^+^ T cell responses in controls and infected patients. Table S1 lists the characteristics of 41 COVID-19–convalescent volunteers. Table S2 lists the characteristics of 20 pre–COVID-19 healthy individuals.

## Supplementary Material

Table S1 lists the characteristics of 41 COVID-19–convalescent volunteers.

Table S2 lists the characteristics of 20 pre–COVID-19 healthy individuals.
